# Sensor-Based Classification of Post-Stroke Motor Impairment Using Fugl-Meyer Lower Extremity Scores

**DOI:** 10.3390/s26144458

**Published:** 2026-07-14

**Authors:** Cristiana Pinheiro, Luís Abreu, Joana Figueiredo, Cristina Cruz, João Cerqueira, Cristina P. Santos

**Affiliations:** 1 Center for MicroElectroMechanical Systems (CMEMS), University of Minho, 4800-058 Guimarães, Portugal; a82888@alunos.uminho.pt (L.A.); joana.figueiredo@dei.uminho.pt (J.F.); cristina@dei.uminho.pt (C.P.S.); 2 LABBELS Associative Laboratory, University of Minho, 4710-057 Braga, Portugal; 3 Department of Physical Medicine and Rehabilitation, Hospital of Braga, 4710-243 Braga, Portugal; cristina@dacruz.pt; 4 Life and Health Sciences Research Institute (ICVS), University of Minho, 4710-057 Braga, Portugal; jcerqueira@med.uminho.pt; 5 Clinical Academic Center (2CA-Braga), Hospital of Braga, 4710-243 Braga, Portugal

**Keywords:** biomarker, clinical scale, sEMG, gait, machine learning, spatiotemporal

## Abstract

This study aims to evaluate multiple feature sets composed of sensor-based biomarkers acquired during walking for the automated estimation of post-stroke motor impairment levels using Fugl-Meyer Lower Extremity Assessment (FMA-LE)-derived classes. Sensor-based walking data from the open-source ARRA dataset were combined with data collected at the Hospital of Braga. Data from 32 post-stroke individuals (FMA-LE motor score: 24 ± 3) were included. A decision tree classifier was evaluated using stratified six-fold cross-validation across different feature sets, including: correlated with motor impairment levels versus full feature sets; spatiotemporal versus surface electromyographic (sEMG) features; inclusion of demographic variables; and the use of data augmentation. The best performance was achieved using correlated sEMG features combined with age, paretic side, and body mass, along with noise-based data augmentation, yielding a validation Matthews Correlation Coefficient (MCC) of 0.85 ± 0.16 and a test MCC of 0.70. sEMG features provided improved classification performance compared to spatiotemporal features, and comparable results were obtained using a reduced subset of muscles. These results demonstrate the feasibility of using sEMG-based features acquired during walking to classify post-stroke motor impairment levels. Feature reduction and inclusion of demographic variables may support efficient model design, while data augmentation may enhance generalization. Further validation in larger and more diverse datasets is required to assess robustness and clinical applicability.

## 1. Introduction

The Fugl-Meyer Assessment (FMA) clinical scale is the primary measure used to evaluate motor impairment in the post-stroke population [1]. The FMA scale characterizes motor impairment in the domains of movement, coordination, speed, and reflex action of upper and lower extremities [2,3,4]. Patients are visually examined by healthcare professionals while performing multiple pre-defined structured physical tasks, resulting in an overall score of motor impairment [2,3,4]. Evaluating these scores is standard in clinical practice and meaningful for diagnostic and therapeutic purposes as they facilitate comparisons of patients and treatments for guiding critical treatment choices for rehabilitation [5].

Although the FMA scale presents excellent inter- and intra-rater reliability, its interpretation may include some subjectivity and dependency on the exposure of clinicians to a wide range of post-stroke impairments [6]. This drawback can be suppressed by exploring objective sensor-based biomarkers correlated with FMA scores once sensor data acquisition provides objective and real-time measurements [7]. Sensor-based biomarkers can be combined with machine learning methods for automated estimation of clinical scores. After being trained by experienced clinicians, the model may be capable of interpreting sensor-based biomarkers to automatically deliver the clinical score, requiring technical training to place sensors and configure equipment that may be achieved with a few supervised sessions as wearable sensor technologies develop.

Current literature has investigated the development of machine learning methods for automatically estimating FMA scores based on sensor-based biomarkers. Both biomechanical and physiological metrics have been investigated [8,9,10,11,12,13]. Tozlu et al. [11] combined demographic, clinical, neurophysiological, and magnetic resonance imaging metrics as features of a random forest, achieving a median r-square of 0.88. Song et al. [10] introduced cellphone movement data while executing FMA-related tasks into a decision tree regression model, succeeding with an average r-square of 0.97. These works only focused on the Fugl-Meyer Upper Extremity Assessment (FMA-LE) scale [8,9,10,11,12,13,14] and, as far as the authors know, there is no study focusing on the Fugl-Meyer Lower Extremity Assessment (FMA-LE) segment of the clinical scale. However, lower extremity motor function impacts activities of daily living such as walking. In this manner, objective measurement of FMA-LE is also essential for monitoring post-stroke recovery progress, as described by Rech et al. [15].

This work aims to evaluate multiple feature sets composed of sensor-based biomarkers acquired during walking for automated estimation of post-stroke motor impairment levels derived from FMA-LE. The findings will contribute to maximizing the use of FMA-LE in clinical practice to support clinical decisions toward prompt rehabilitation for post-stroke patients. This work uses both collected (for testing) and open-source datasets (for training, validating, and testing).

## 2. Materials and Methods

### 2.1. Participants

Data from an open-source ARRA dataset (acquired at Medical University of South Carolina; Section S1.1 of Supplementary Materials) [16] were merged with data acquired at the Hospital of Braga, producing a larger dataset with 32 post-stroke subjects (12 female, 53 ± 8 years, 80 ± 8 kg, 24 ± 3 FMA-LE motor score, 16 paretic left side). All eligible subjects gave their informed consent for inclusion before they participated in the studies [16].

Data acquired at the Hospital of Braga followed the Declaration of Helsinki and the protocol approval by the Ethics Committee of CEHB 157_2021 (“Comissão Ética Hospital Braga 157_2021”). We collected data from 5 post-stroke subjects (3 female, 45 ± 18 years, 69 ± 15 kg, 24 ± 5 FMA-LE motor score, 2 paretic left side, 12 ± 7 months after the stroke, 3 ischemic), using the following subject inclusion criteria: (1) history of single unilateral stroke; (2) lower limb muscle spasticity medically controlled (i.e., cases where lower-limb spasticity was clinically managed by the treating physician); and (3) able to complete the 10 m walk test. Subjects were excluded according to the following exclusion criteria: (1) significant cognitive impairment limiting their active participation in the study; (2) neurological, orthopedic, cardiac, or respiratory disease affecting locomotion; and (3) aphasia. These data were acquired to be used as a separate test set to evaluate model generalization.

### 2.2. Experimental Protocol and Data Collection

At the Medical University of South Carolina, the participants were instructed to perform three 30 s trials walking on an instrumented treadmill at their self-selected speed. Surface electromyography (sEMG) data were acquired while walking at 1000 Hz (Motion Lab Systems, Baton Rouge, LA, USA) from 8 muscles in the paretic limb (tibialis anterior, soleus, medial gastrocnemius, vastus medialis, rectus femoris, medial hamstring, lateral hamstring, and gluteus medius). sEMG trials were filtered with a zero-lag, fourth-order, high-pass Butterworth filter at 40 Hz, rectified, and low-pass filtered at 4 Hz. Filtered trials were segmented into gait cycles and then averaged per trial, producing 71 averaged curves. Averaged curves were normalized to the peak value of each trial. Kinematic data were also recorded at 120 Hz from a 12-camera motion capture system (PhaseSpace, San Leandro, CA, USA) using reflective markers placed on lower limbs and torso following a modified Helen Hayes marker set. Moreover, the treadmill’s force plates allowed the acquisition of 3D ground reaction force at 2000 Hz. Kinematic and ground reaction force data were filtered using a fourth-order Savitzky–Golay least-square polynomial filter and resampled at 100 Hz.

At the Hospital of Braga, each subject performed three 10 m overground walking trials at their self-selected speed. sEMG data were acquired while walking at 2148 Hz (Trigno sEMG system, Delsys, Natick, MA, USA) from 4 muscles in the paretic limb (tibialis anterior, lateral gastrocnemius, rectus femoris, and biceps femoris). Electrode placement followed the SENIAM recommendations, appropriate skin preparation was performed prior to sensor placement, and all walking trials were supervised to ensure proper sensor attachment and consistent data acquisition. Data were post-processed to match post-processing from the ARRA dataset [16], resulting in a total of 13 normalized averaged curves.

The Fugl-Meyer Assessment was carried out by an expert with a biomedical background to determine the FMA-LE clinical motor score (Section S1.2 of Supplementary Materials). This corresponds to a single cross-sectional clinical assessment per participant, not a longitudinal evaluation across rehabilitation stages or multiple sessions.

### 2.3. Feature Extraction

The following widely used frequency domain features were extracted from the sEMG curves (from both ARRA and Hospital of Braga datasets) overcoming possible noise in the signal by extracting its stable characteristics: mean (MNF), median (MDF), and peak (PKF) power frequencies [17]. Mean frequency was calculated as a ratio between the product sum of the sEMG power spectrum intensity with the frequency, and the sum of the power spectrum intensity. Median frequency was determined as the frequency at which the sEMG power spectrum intensity was divided into two regions with equal amplitude, while peak frequency was the frequency at which the maximum power occurred [17].

The ARRA dataset provided spatiotemporal parameters, derived from kinematic and ground reaction force data, for each gait cycle, including non-paretic and paretic step length (m), stride length (m), stride time (s), and cadence (steps/min). Demographic features including gender (male and female defined as 1 and 0, respectively), age (years), body mass (kg), and paretic side (right and left paretic sides defined as 1 and 0, respectively), were also registered.

### 2.4. Data Labelling

Participants were classified with a low, mid, or high motor impairment level based on FMA-LE clinical motor scores. Smith et al. [18] reported the FMA-LE motor score for a sample of 93 post-stroke patients able to walk independently as ranging from 9. Thus, high motor impairment was automatically considered for patients unable to walk (FMA-LE motor score < 9 according to Smith et al. [18]). Mid (label 0) and low (label 1) motor impairment levels were defined for 9 ≤ FMA-LE < 21 and FMA-LE ≥ 21, respectively, according to Kwong et al. [19]. According to the inclusion criteria of both experimental protocols, patients with high motor impairment were not included in this study. Ten participants were labelled with mid impairment (1 from the Hospital of Braga and 9 from the ARRA dataset) and 22 participants with low impairment (4 from the Hospital of Braga and 18 from ARRA dataset).

Spearman’s correlation coefficients, r, were calculated between FMA-LE-derived classes (mid and low motor impairment levels) and sEMG or spatiotemporal features. The correlation strength was interpreted as moderate if r=0.40−0.69 and strong if r≥0.7 [20]. The correlated features were identified by presenting a moderate to strong correlation (r≥0.4).

### 2.5. Data Splitting

Data from the ARRA dataset (27 subjects, 71 trials) were split at subject level into training and test sets using an 80–20% random stratified split according to motor impairment class (mid/low), ensuring class stratification in both partitions.

Within the training set (57 trials), this configuration resulted in 19 trials (33%) being classified as mid impairment and 38 trials (67%) being classified as low impairment. The training set was used exclusively for model development and was further evaluated using StratifiedGroupKFold cross-validation with 6 folds. This approach ensured that all trials from a given subject were assigned to the same fold, preventing subject-level data leakage between training and validation partitions. Stratification was applied at the subject level to preserve the distribution of motor impairment classes (mid/low) across folds.

For testing, two different datasets were used, including (a) only ARRA dataset trials (3 trials as mid impairment and 11 trials as low impairment) and (b) trials from ARRA merged with the ones from the Hospital of Braga—the hybrid test set (6 trials as mid impairment and 21 trials as low impairment).

### 2.6. Training Dataset Preparation

Training dataset preparation methods were applied, namely adding noise or Synthetic Minority Over-sampling Technique (SMOTE) methods. White or pink noise was added to all features from the training set as a data augmentation technique to foster model generalization ability [21] and to balance the classes. White noise characterizes the thermal noise of sEMG signals created by the amplification circuits of the sensors [22]. On the other hand, pink noise is related to the electrochemical noise of sEMG signals created by the interface between the skin and electrodes [22].

White and pink noise data were calculated following a flat and linear frequency spectrum [22], respectively, and added to the existing training dataset considering an amplitude factor of 0.1. This factor was chosen to simulate realistic signal perturbations without significantly altering the underlying sEMG features, in line with common practices in data augmentation [21]. Nine noisy trials were randomly added and replaced existing ones for the mid and low motor impairment classes, respectively. We selected nine trials as this corresponds to less than half (32%) of the trials belonging to the mid-performance class; thus, the resulting dataset was not largely affected by the synthetic noise. Moreover, the imbalance level between the classes decreased, resulting in 28 (42%) and 38 trials (58%) for mid and low motor impairment classes, respectively. This procedure resulted in three training sets: (a) without noisy samples, (b) with white noisy samples, and (c) with pink noisy samples. The same set of randomly selected training trials was used across the white and pink noise conditions to ensure a fair comparison between the two augmentation strategies.

A fourth training set was created by applying SMOTE. SMOTE was executed considering 5 neighbours (as in [23]) and the same imbalance proportion as the noisy training sets (38 low per 28 mid impairment samples) to allow comparison between dataset preparation methods.

### 2.7. Machine Learning Model

**Figure 1:**
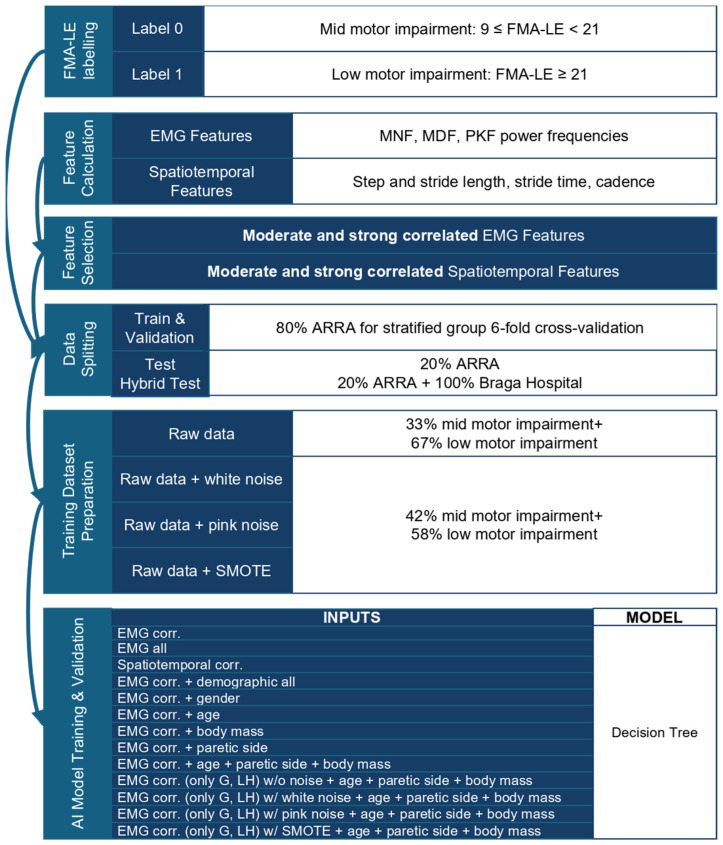
Diagram of the methodological workflow (“corr.”: moderate and strong correlated, “G”: gastrocnemius, “LH”: lateral hamstring).

We evaluated the performance of a state-of-the-art decision tree classifier [8,10,11] trained with multiple feature sets to explore which are the most suitable for classifying the post-stroke motor impairment levels. Figure 1 summarizes the methodological workflow of this work.

As a preliminary study, the decision tree classifier was considered a suitable choice due to its interpretability which allows healthcare professionals to make informed decisions based on the model’s estimation, ability to handle both continuous (e.g., sensor-based features) and categorical (e.g., demographic features) data, and its capacity to capture non-linear relationships between features [24]. Moreover, a decision tree is robust to outliers, scalable for larger datasets, and adaptable to imbalanced data [24]. Regressor performance was not evaluated due to the limited open-source data from post-stroke patients covering the full-range FMA-LE. All algorithms were imported from the Scikit-Learn 1.3 Python 3.9 library.

Decision tree classifier hyperparameters include the function to measure the quality of a split, “criterion”; the strategy used to choose the split at each node, “splitter”; the maximum depth of the tree, “max_depth”; the minimum number of samples required to split an internal node, “min_samples_split”; the minimum number of samples required to be at a leaf node, “min_samples_leaf”; the number of features to consider when looking for the best split, “max_features”; the maximum number of leaf nodes, “max_leaf_nodes”; weights associated with classes, “class_weight”; and a complexity parameter used for minimal cost-complexity pruning, “ccp_alpha”. The optimization of these hyperparameters is shown in the intervals of Table S1 in Supplementary Materials. A grid search method was implemented to find the best hyperparameters for the model with each training set (Table S2 in Supplementary Materials).

Model performance was evaluated with the Matthews Correlation Coefficient (MCC), F1 score, recall, and confusion matrix metrics, averaged over the 6 folds of cross-validation (Section S1.3 of Supplementary Materials). The maximum score (value of 1) represents a perfect classification. The best hyperparameters were selected to maximize MCC once it produced an informative and truthful score in evaluating binary classification [25]. To address the known limitations of decision trees, overfitting was evaluated by comparing training and validation MCC while increasing model complexity (‘max depth’).

### 2.8. Statistical Analysis

The non-parametric Wilcoxon signed-rank test was used with the SciPy Python library to evaluate the models’ performance using a confidence level of 0.05 and considering model performance metrics in each fold. The following null hypotheses were analysed: there are no significant differences regarding the model performance metric between (a) correlated vs. all sEMG features; (b) correlated sEMG vs. spatiotemporal features; (c) correlated sEMG features vs. combination with demographic features; (d) correlated sEMG features from gastrocnemius, vastus, gluteus, and lateral hamstring muscles vs. correlated sEMG features from only gastrocnemius and lateral hamstring muscles (matching the correlated muscles from both ARRA and Hospital of Braga); and (e) noise-free vs. noisy augmented or SMOTE features. The null hypotheses were iteratively defined based on the feature set that achieved the best performance in the previous comparison, which was then used as the reference condition for subsequent tests.

## 3. Results

The resulting moderately to strongly correlated sEMG features (Figure S1 in Supplementary Materials) comprised all sEMG features from paretic gluteus medius (GM, 0.43≤r≤0.49) and median and peak frequencies from medial gastrocnemius (MG, 0.46≤r≤0.53), vastus medialis (VM, 0.45≤r≤0.50), and lateral hamstring (LH, 0.46≤r≤0.48). Regarding spatiotemporal features (Figure S2 in Supplementary Materials), the moderately to strongly correlated features included paretic and non-paretic stride length (r=0.44), non-paretic step length (r=0.57), paretic stride time (r=−0.51), and cadence (r=0.51).

**Figure 2:**
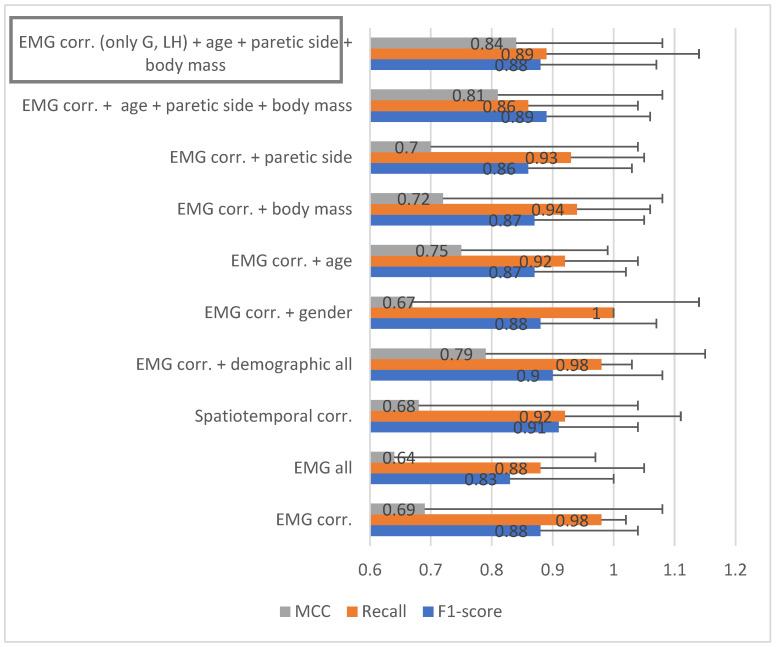
Mean and standard deviation of F1, recall, and MCC validation scores using different features: all sEMG features, correlated (corr.) sEMG features, correlated spatiotemporal features, and correlated sEMG features combined with demographic features (age, gender, body mass, paretic side). A decision tree classifier was used. The result with the maximum MCC validation score is highlighted. G and LH define gastrocnemius and lateral hamstring muscles, respectively.

Figure 2 exhibits mean and standard deviation F1, recall, and MCC validation scores considering the multiple feature sets: all sEMG features, correlated sEMG features, correlated spatiotemporal features, or correlated sEMG features combined with demographic features. Correlated sEMG features achieved higher training and validation MCC scores than correlated spatiotemporal features (Table S3 from Supplementary Materials), and were significantly different to the training scores (*p*-value = 0.03). Furthermore, the use of correlated sEMG features revealed higher validation scores than using all sEMG features. However, training and validation scores were not considered significantly different (*p*-value ≥ 0.28). A higher MCC validation score was observed for the combination of sEMG features with age, body mass, and paretic side. Individually, although not significantly different, age, body mass, and paretic side increased the average and decreased the standard deviation of the MCC validation score (*p*-value ≥ 0.14). In contrast, gender decreased the average and increased the standard deviation.

Despite the significant decrease in the training scores (*p*-value = 0.03), the MCC validation score was not significantly affected by using sEMG features from only the gastrocnemius and lateral hamstring muscles (*p*-value ≥ 0.32). Furthermore, the combination of correlated sEMG features from only gastrocnemius and lateral hamstring muscles with age, body mass, and paretic side demographic features resulted in the MCC validation score of 0.84 ± 0.24. This combination of features was used for testing the model with the hybrid test set (Figure S5 in Supplementary Materials).

**Figure 3:**
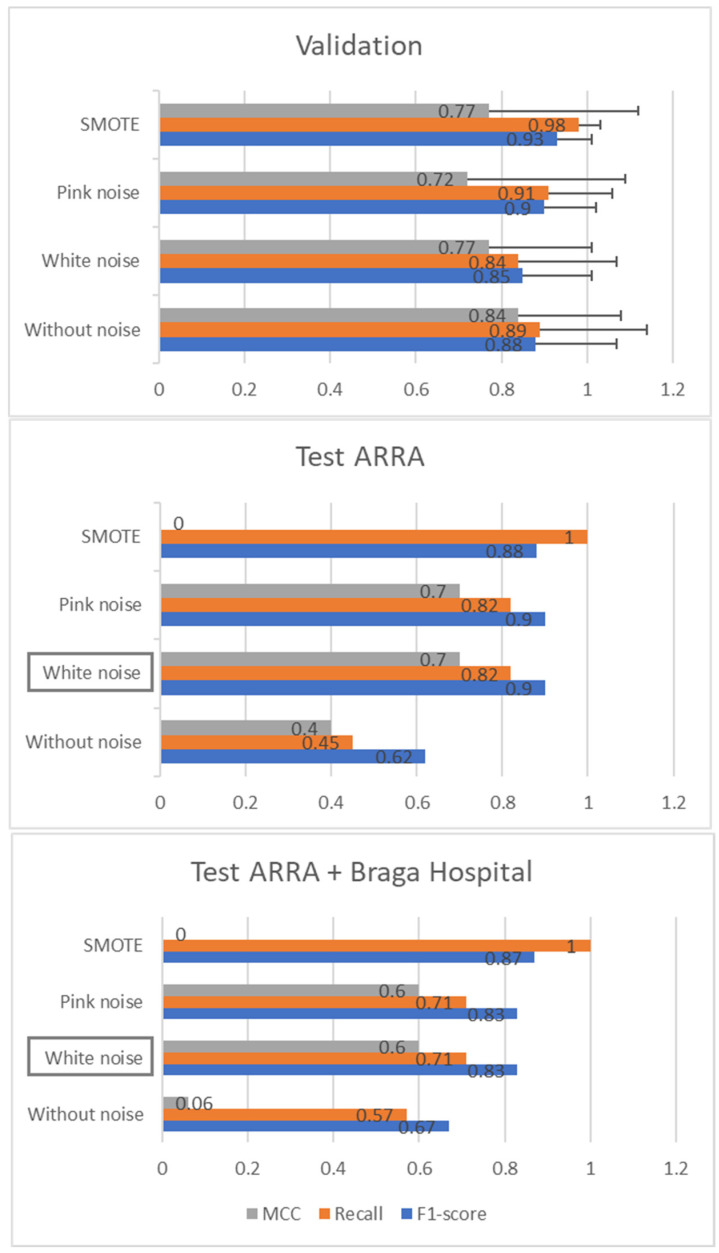
Mean and standard deviation of F1, recall, and MCC validation and test scores (ARRA test set or hybrid test set from ARRA and Braga Hospital) using features after different data preparation methods: correlated sEMG features (only gastrocnemius and lateral hamstring muscles) combined with demographic features (age, body mass, paretic side) and the addition of noisy (white or pink noise) or SMOTE samples. A decision tree classifier was used. The result with the maximum MCC test score is highlighted. The best hyperparameters found were the gini function, random split strategy, 4 maximum depths of the tree, a minimum of 2 samples to split an internal node and 4 samples required to be at a leaf node, square of features for the number of features to consider for the best split, 8 maximum leaf nodes, balanced weights, and complexity parameter equal to 0.

Figure 3 shows the mean and standard deviation values of F1, recall, and MCC validation and test scores considering correlated sEMG features with the addition of noisy or SMOTE samples. The sEMG features (only from gastrocnemius and lateral hamstring) were combined with age, paretic side, and body mass demographic features. By analysing Figure 3, a higher MCC validation score appeared for the non-noisy features. White noise presented a slightly higher MCC validation score (MCC 0.77 ± 0.24) compared with pink noise (MCC 0.72 ± 0.37) and was similar to SMOTE features (MCC 0.77 ± 0.35) but was not significantly different from the non-noisy features (*p*-value ≥ 0.46) (Table S4 in Supplementary Materials). However, the highest test score appeared when using both white and pink noisy samples in training data preparation, and this was considered to be the best model found (Figures S3–S5 in Supplementary Materials). The hybrid dataset presented a lower test score than the ARRA dataset.

## 4. Discussion

Sensors provide objective real-time data, such as muscle activity, which can be difficult to assess through visual physical examination, especially to distinguish close impairment levels [7]. Machine learning algorithms may detect complex patterns in sensor data that might not be immediately apparent, enabling more informed evaluations of motor impairment [24]. This work evaluates the performance of a decision tree classifier with multiple sensor-based features acquired during walking to estimate post-stroke motor impairment defined by FMA-LE-derived classes.

Although spatiotemporal parameters revealed an individual higher correlation with FMA-LE-derived classes (0.44≤|r|≤0.57), sEMG group features (0.43≤|r|≤0.53) were more suitable to achieve better model performance. In line with the literature, physiological features often demonstrated higher performance scores than biomechanical features for estimating FMA-UE [8,9,11].

Correlated sEMG features were shown to be sufficient to achieve higher model performance scores when compared to the use of all sEMG features, revealing that training and validation scores were statistically similar between them. In this context, feature selection based on correlation analysis allowed a reduction in input dimensionality without a significant loss in performance, consistent with prior work using correlation analysis to identify informative and non-redundant features [26].

Considering the correlated sEMG features, the results allowed a preliminary exploration of sensor placement requirements within this experimental context. The findings indicated that sEMG signals from the medial gastrocnemius, vastus medialis, lateral hamstring, and gluteus medius showed stronger association with FMA-LE classes, whereas signals from the tibialis anterior, soleus, rectus femoris, and medial hamstring exhibited weaker correlations. Furthermore, model performance was not significantly affected when only sEMG features from the medial gastrocnemius and lateral hamstring were used. These results suggest that, within this dataset, FMA-LE classification during walking may be achievable using sEMG data from a reduced set of muscles. This reduction could potentially decrease subject preparation time for sensor placement. However, this observation is specific to the present model and dataset and should be validated in broader populations and experimental conditions. Moreover, we acknowledge that sEMG recordings remain susceptible to crosstalk between adjacent muscles. Therefore, the observed importance of individual muscles should be interpreted with caution, as it may partly reflect activity from neighbouring muscles.

Although a reduced number of sensors may simplify the acquisition protocol, technical training for healthcare professionals, including supervised sessions on proper sensor placement and equipment configuration, is still likely to be required to ensure reliable data acquisition and accurate model input [27]. Advances in wearable sensor technologies may further reduce the complexity of these procedures over time. Future research should evaluate the extent of training required to achieve consistent and reliable measurements, particularly in comparison with the expertise needed for traditional clinical-scale assessments [4]. Furthermore, future studies should systematically investigate the trade-off between sensor reduction and physiological completeness to ensure that clinically relevant compensatory strategies are not overlooked.

The addition of demographic features improved the model performance, consistent with previous findings from Tozlu et al. [11]. Specifically, age, body mass, and paretic side were associated with higher classification scores in this work. These observations are consistent with prior literature indicating that long-term functional recovery after stroke may vary with age, body mass index, and side of impairment, with older age associated with slower or reduced recovery [28], higher body mass index linked to less favourable rehabilitation outcomes [29], and differences in recovery trajectories observed between hemiparetic sides [30]. In addition, in the present work, these variables should be interpreted as contributing to model discrimination rather than as direct indicators of recovery mechanisms. Although previous studies report that female patients may experience greater challenges in post-stroke recovery [31], gender did not show a positive contribution to FMA-LE classification performance in this dataset, suggesting that its predictive value may be limited or context-dependent.

The data augmentation did not significantly improve validation scores. However, the addition of white and pink noise data during training was associated with higher test performance, increasing MCC scores from 0.40 to 0.70. This suggests that noise-based augmentation may have enhanced model robustness to variability in unseen data. Similar findings have been reported in sensor-based machine learning studies, where data augmentation techniques were shown to improve generalization in time-series signals collected from movement disorders [21].

Overall, the best model achieved validation F1, recall, and MCC scores of 0.85 ± 0.16, 0.84 ± 0.23, and 0.77 ± 0.24, respectively. The selected configuration (max depth = 4) resulted in a training MCC of 0.77 and a validation MCC of 0.70 (Figure S3 in Supplementary Materials), indicating a relatively small performance gap and no strong evidence of overfitting within the cross-validation framework used. Previous studies, such as those by Song et al. [10] and Riahi et al. [12], reported strong predictive performance for FMA-UE estimation using alternative data modalities, including smartphone-based movement data and resting-state electroencephalography. However, these studies evaluated regression performance (e.g., R^2^), which is not directly comparable to the classification framework adopted in the present work.

The model also achieved acceptable performance on both the ARRA (MCC = 0.70) and hybrid datasets (MCC = 0.60). The lower performance observed in the hybrid test set may reflect reduced generalization capability under dataset shift conditions. This could be related to differences in acquisition protocols, including overground versus treadmill walking, variability in examiner experience, and differences in demographic distributions such as age and body mass (Braga dataset: 45 ± 18 years, 69 ± 15 kg; ARRA dataset: 60 ± 12 years, 92 ± 19 kg). However, these factors cannot be isolated in the present study and therefore their individual contribution cannot be determined. Although data collection constraints such as equipment availability, patient recruitment, and clinical scheduling are common in applied biomedical studies, future work should control or explicitly model these sources of variability to improve generalizability. In addition, we acknowledge the lack of detailed clinical metadata for the ARRA dataset (e.g., stroke subtype and time since stroke) and that spasticity was not quantitatively assessed using a standardized clinical scale in this study (e.g., the Modified Ashworth Scale) and was therefore not included in the present analysis. Future studies should incorporate detailed clinical metadata including standardized measures of spasticity to further investigate its influence on impairment classification.

The performance of the decision tree classifier used in this study was likely constrained by the limited sample size and variability of the dataset, which may affect model stability and generalizability. Further validation using larger, multi-site datasets collected under heterogeneous clinical settings is needed to better quantify model robustness and generalizability. The results highlight the need for larger and more diverse datasets of post-stroke patients using wearable sensors [10,32]. The availability of such datasets, particularly in open-access formats, would support more robust model training and enable evaluation across heterogeneous populations, thereby improving generalizability and potential clinical applicability. The contribution of demographic variables should also be explored in greater depth to determine whether they capture inter-subject variability not represented by sensor-derived features.

Despite these limitations, this work demonstrates the feasibility of using sEMG-based features acquired through wearable sensors during walking to estimate FMA-LE-derived classes with a decision tree model. Future research should further explore clinician-in-the-loop approaches to enhance the interpretability and clinical utility of the proposed framework. Although the decision tree provides interpretability at the feature-decision level, linking these features to specific neurophysiological mechanisms (e.g., compensatory movements) requires further investigation. We also agree that longitudinal prediction of FMA-LE evolution represents a highly relevant and clinically valuable research direction. Future studies could investigate sequence-based models that preserve the temporal structure of sEMG and kinematic signals to determine whether additional clinically meaningful information can be extracted beyond the current feature-based representation.

## 5. Conclusions

This study demonstrates the feasibility of using sEMG-based features acquired through wearable sensors during walking to classify post-stroke motor impairment through FMA-LE-derived classes. The results show that comparable model performance can be achieved using a reduced subset of muscles and that the inclusion of demographic features may enhance classification performance. In addition, noise-based data augmentation was associated with improved test performance, suggesting a potential role in enhancing model generalization. However, model performance remained sensitive to dataset variability, highlighting the impact of dataset shift on generalization. These findings support the potential of wearable sensor-based approaches for assisting motor impairment assessment, while emphasizing the need for validation in larger and more diverse populations.

## Data Availability

The ARRA dataset is publicly available, as described in the original publication. The clinical dataset collected at the Hospital of Braga is not publicly available due to the limited size of the cohort and to avoid potential re-identification risks. Data supporting the findings of this study are available from the corresponding author upon reasonable request.

## Supplementary Materials

The following supporting information can be downloaded at: https://www.mdpi.com/article/10.3390/s26144458/s1, Figure S1: Boxplot of the correlated sEMG features per class; Figure S2: Boxplot of spatiotemporal features per class; Table S1: Intervals for optimizing hyperparameters; Table S2: Best hyperparameters found for each input; Figure S3: Training and validation score curves per model complexity; Figure S4: Confusion matrix; Figure S5: Tree diagram; Table S3: F1, recall, and MCC scores achieved by decision tree for training and validation datasets using different inputs; Table S4: F1, recall, and MCC scores for training, validation, and test sets using inputs after different data preparation methods.

## Abbreviations

The following abbreviations are used in this manuscript:

sEMG	Surface electromyography
FMA	Fugl-Meyer Assessment
FMA-LE	Fugl-Meyer Lower Extremity Assessment
FMA-UE	Fugl-Meyer Upper Extremity Assessment
MCC	Matthews Correlation Coefficient
MDF	Median power frequency
MNF	Mean power frequency
PKF	Peak power frequency
SMOTE	Synthetic Minority Over-sampling Technique

## References

[1] Bushnell C., Bettger J.P., Cockroft K.M., Cramer S.C., Edelen M.O., Hanley D., Katzan I.L., Mattke S., Nilsen D.M., Piquado T. (2015). Chronic Stroke Outcome Measures for Motor Function Intervention Trials. Circ. Cardiovasc. Qual. Outcomes.

[2] Duncan P.W., Propst M., Nelson S.G. (1983). Reliability of the Fugl-Meyer Assessment of Sensorimotor Recovery Following Cerebrovascular Accident. Phys. Ther..

[3] Sanford J., Moreland J., Swanson L.R., Stratford P.W., Gowland C. (1993). Reliability of the Fugl-Meyer Assessment for Testing Motor Performance in Patients Following Stroke. Phys. Ther..

[4] Sullivan K.J., Tilson J.K., Cen S.Y., Rose D.K., Hershberg J., Correa A., Gallichio J., McLeod M., Moore C., Wu S.S. (2011). Fugl-Meyer Assessment of Sensorimotor Function After Stroke. Stroke.

[5] Harrison J.K., McArthur K.S., Quinn T.J. (2013). Assessment Scales in Stroke: Clinimetric and Clinical Considerations. Clin. Interv. Aging.

[6] Gladstone D.J., Danells C.J., Black S.E. (2002). The Fugl-Meyer Assessment of Motor Recovery after Stroke: A Critical Review of Its Measurement Properties. Neurorehabilit. Neural Repair.

[7] Routson R.L., Kautz S.A., Neptune R.R. (2014). Modular Organization across Changing Task Demands in Healthy and Poststroke Gait. Physiol. Rep..

[8] Julianjatsono R., Ferdiana R., Hartanto R. (2017). High-Resolution Automated Fugl-Meyer Assessment Using Sensor Data and Regression Model. Proceedings of the 2017 3rd International Conference on Science and Technology—Computer (ICST).

[9] Gebruers N., Truijen S., Engelborghs S., De Deyn P.P. (2014). Prediction of Upper Limb Recovery, General Disability, and Rehabilitation Status by Activity Measurements Assessed by Accelerometers or the Fugl-Meyer Score in Acute Stroke. Am. J. Phys. Med. Rehabil..

[10] Song X., Chen S., Jia J., Shull P.B. (2019). Cellphone-Based Automated Fugl-Meyer Assessment to Evaluate Upper Extremity Motor Function After Stroke. IEEE Trans. Neural Syst. Rehabil. Eng..

[11] Tozlu C., Edwards D., Boes A., Labar D., Tsagaris K.Z., Silverstein J., Pepper Lane H., Sabuncu M.R., Liu C., Kuceyeski A. (2020). Machine Learning Methods Predict Individual Upper-Limb Motor Impairment Following Therapy in Chronic Stroke. Neurorehabilit. Neural Repair.

[12] Riahi N., Vakorin V.A., Menon C. (2020). Estimating Fugl-Meyer Upper Extremity Motor Score From Functional-Connectivity Measures. IEEE Trans. Neural Syst. Rehabil. Eng..

[13] Zhou Y.M., Raman N., Proietti T., Arnold J., Pathak P., Pont-Esteban D., Nuckols K., Rishe K., Doshi-Velez F., Lin D. (2025). Estimating Upper Extremity Fugl-Meyer Assessment Scores From Reaching Motions Using Wearable Sensors. IEEE J. Biomed. Heal. Inform..

[14] Chen S., Lin X., Fu J., Qian Y., Chen Z., Huang Z., Liu Q., Lu X., Jia J. (2023). Prediction of the Hand Function Part of the Fugl-Meyer Scale after Stroke Using an Automatic Quantitative Assessment System. Brain-X.

[15] Rech K.D., Salazar A.P., Marchese R.R., Schifino G., Cimolin V., Pagnussat A.S. (2020). Fugl-Meyer Assessment Scores Are Related With Kinematic Measures in People with Chronic Hemiparesis after Stroke. J. Stroke Cerebrovasc. Dis..

[16] Kautz S.A., Neptune R.R. Medical University of South Carolina Stroke Data (ARRA). https://www.icpsr.umich.edu/web/ICPSR/studies/37122.

[17] Oskoei M.A., Hu H. (2008). Support Vector Machine-Based Classification Scheme for Myoelectric Control Applied to Upper Limb. IEEE Trans. Biomed. Eng..

[18] Smith M.-C., Barber A.P., Scrivener B.J., Stinear C.M. (2022). The TWIST Tool Predicts When Patients Will Recover Independent Walking After Stroke: An Observational Study. Neurorehabilit. Neural Repair.

[19] Kwong P.W.H., Ng S.S.M. (2019). Cutoff Score of the Lower-Extremity Motor Subscale of Fugl-Meyer Assessment in Chronic Stroke Survivors: A Cross-Sectional Study. Arch. Phys. Med. Rehabil..

[20] Dancey C.P., Reidy J. (2007). Statistics Without Maths for Psychology.

[21] Um T.T., Pfister F.M.J., Pichler D., Endo S., Lang M., Hirche S., Fietzek U., Kulić D. (2017). Data Augmentation of Wearable Sensor Data for Parkinson’s Disease Monitoring Using Convolutional Neural Networks. Proceedings of the 19th ACM International Conference on Multimodal Interaction, Glasgow, UK, 13–17 November 2017.

[22] Boyer M., Bouyer L., Roy J.-S., Campeau-Lecours A. (2023). Reducing Noise, Artifacts and Interference in Single-Channel EMG Signals: A Review. Sensors.

[23] Chawla N.V., Bowyer K.W., Hall L.O., Kegelmeyer W.P. (2002). SMOTE: Synthetic Minority Over-Sampling Technique. J. Artif. Intell. Res..

[24] Breiman L. (1984). Classification and Regression Trees.

[25] Chicco D., Jurman G. (2020). The Advantages of the Matthews Correlation Coefficient (MCC) over F1 Score and Accuracy in Binary Classification Evaluation. BMC Genom..

[26] Kumar S., Chong I. (2018). Correlation Analysis to Identify the Effective Data in Machine Learning: Prediction of Depressive Disorder and Emotion States. Int. J. Environ. Res. Public Health.

[27] Hilty D.M., Armstrong C.M., Edwards-Stewart A., Gentry M.T., Luxton D.D., Krupinski E.A. (2021). Sensor, Wearable, and Remote Patient Monitoring Competencies for Clinical Care and Training: Scoping Review. J. Technol. Behav. Sci..

[28] Yoo J., Hong B., Jo L., Kim J.-S., Park J., Shin B., Lim S. (2020). Effects of Age on Long-Term Functional Recovery in Patients with Stroke. Medicina.

[29] Leszczak J., Czenczek-Lewandowska E., Przysada G., Baran J., Weres A., Wyszyńska J., Mazur A., Kwolek A. (2019). Association Between Body Mass Index and Results of Rehabilitation in Patients After Stroke: A 3-Month Observational Follow-Up Study. Med. Sci. Monit..

[30] Bindawas S.M., Mawajdeh H.M., Vennu V.S., Alhaidary H.M. (2017). Functional Recovery Differences after Stroke Rehabilitation in Patients with Uni- or Bilateral Hemiparesis. Neurosciences.

[31] Kim J.-S., Lee K.-B., Roh H., Ahn M.-Y., Hwang H.-W. (2010). Gender Differences in the Functional Recovery after Acute Stroke. J. Clin. Neurol..

[32] Sanchez N. Stroke Initiative for Gait Data Evaluation (STRIDE), United States, 2012–2020. https://www.icpsr.umich.edu/web/ICPSR/studies/38002#.

