# Supplementary material for: Sensor-Based Classification of Post-Stroke Motor Impairment Using Fugl-Meyer Lower Extremity Scores
Source: Sensors (Basel). 2026 Jul 14;26(14):4458. doi: 10.3390/s26144458 (PMC13418665; doi:10.3390/s26144458)
Supplement: Supplementary file 1 [file sensors-26-04458-s001.zip › sensors-4346094-supplementary.pdf]

## Supplementary material

## 1. Methods

### 1.1 Participants from ARRA dataset

The open-source ARRA dataset[1] includes 27 post-stroke participants (9 female, 60±12 years, 92±19 kg, 23±7 FMA-LE score, 14 paretic left side, >6 months after the stroke) recruited from the Medical University of South Carolina (USA). The following subject inclusion criteria were applied: 1) a history of single unilateral stroke more than six months before the study, 2) ability to walk over 10 m on a level surface, 3) free of significant lower extremity joint pain, contractures, range of motion limitations, and significant sensory deficits, 4) walk daily at home, 5) with no severe cognitive deficits, 6) no significant cardiovascular impairments contraindicative to walking.

### 1.2. FMA

FMA scores range from 0, matching total hemiparetic motor impairment, to 100 (66 score points for upper extremity and 34 score points for lower extremity), indicating no motor impairment (healthy condition)[2–4]. FMA-LE range is obtained by summing the following subscale scores: reflex activity (0-2 score), volitional movement within synergies (0-14 score), volitional movement mixing synergies (0-4 score), volitional movement with little or no synergy (0-4 score), normal reflex activity (0-2 score), and subscale of coordination and speed (0-6 score)[2–4].

### 1.3. ML Evaluation

MCC ranges between -1 and 1, where 1 represents a perfect prediction, 0 indicates a random prediction, and -1 denotes a total disagreement between the predictions and the true labels. MCC provides a balanced measure of the classifier's performance. F1-score and recall range between 0 and 1, with the maximum score being a perfect prediction. In contrast with MCC, the F1-score is strongly affected by the majority class[5]. Recall represents the ability of the classifier to find all the positive samples (high performance samples). These metrics were also used with the test set.


1. Kautz, S.A.; Neptune, R.R. Medical University of South Carolina Stroke Data (ARRA) Available online: https://www.icpsr.umich.edu/web/ICPSR/studies/37122.
2. Duncan, P.W.; Propst, M.; Nelson, S.G. Reliability of the Fugl-Meyer Assessment of Sensorimotor Recovery Following Cerebrovascular Accident. *Phys. Ther.* **1983**, *63*, 1606–1610, doi:10.1093/ptj/63.10.1606.
3. Sanford, J.; Moreland, J.; Swanson, L.R.; Stratford, P.W.; Gowland, C. Reliability of the Fugl-Meyer Assessment for Testing Motor Performance in Patients Following Stroke. *Phys. Ther.* **1993**, *73*, 447–454, doi:10.1093/ptj/73.7.447.
4. Sullivan, K.J.; Tilson, J.K.; Cen, S.Y.; Rose, D.K.; Hershberg, J.; Correa, A.; Gallichio, J.; McLeod, M.; Moore, C.; Wu, S.S.; et al. Fugl-Meyer Assessment of Sensorimotor Function After Stroke. *Stroke* **2011**, *42*, 427–432, doi:10.1161/STROKEAHA.110.592766.
5. Buhl, N. F1 Score in Machine Learning.

## *2.* Results

Figure S1 shows the boxplot of the correlated EMG features per class considering the (a) ARRA dataset and (b) data acquired at the Hospital of Braga. The mid impairment class presents lower values of average median (MDF), mean (MNF), and peak (PKF) power frequencies for both ARRA and Hospital of Braga datasets.

Figure S2 shows the boxplot of spatiotemporal features per class considering the ARRA dataset. The mid impairment class shows lower values of stride length (m), step length (m), and cadence (steps/min), but higher values of stride time (s) in comparison with low impairment class.

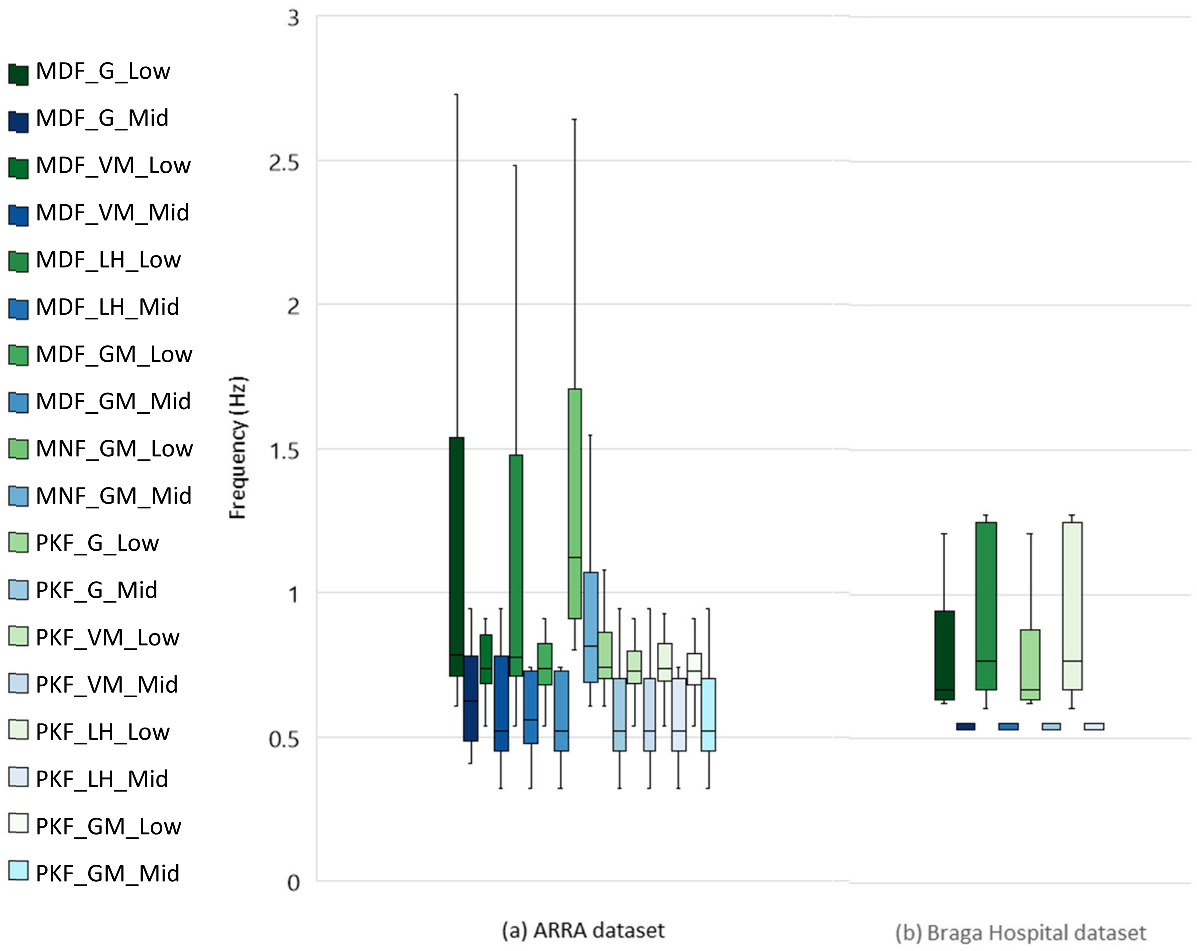


Figure S1. Boxplot of the correlated EMG features per class (low, mid impairment level) considering the (a) ARRA dataset and (b) data acquired at Hospital of Braga.

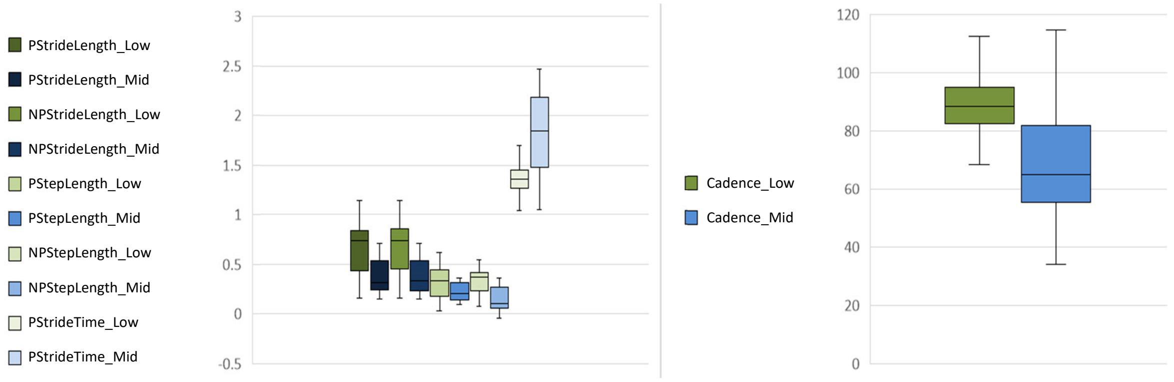


Figure S2. Boxplot of spatiotemporal features per class (low, mid impairment level) considering the ARRA dataset.

Table S1. Intervals of grid search for Decision Tree model

| **Hyperparameter** | **Grid search** |
|---|---|
| criterion | gini, entropy, log_loss |
| splitter | best, random |
| max_depth | 1, 2, 3, 4, 5, 6, 8, 12, 24, 30, None |
| min_samples_split | 1, 2, 3, 4, 6 |
| min_samples_leaf | 1, 2, 3 |
| max_features | auto, sqrt, log2, None |
| max_leaf_nodes | None, 4, 8, 12 |
| class_weight | balanced |
| ccp_alpha | EMG features: 0.0, 0.01, 0.07, 0.08, 0.26<br>Spatiotemporal features: 0.0, 0.03, 0.04, 0.08, 0.26 |

The best hyperparameters found for each input are presented in Table S2.

Table S2. Best hyperparameters for Decision Tree using different input

| **Input** | **Best hyperparameters** |
|---|---|
| Correlated spatiotemporal features | criterion=gini,<br>splitter=random,<br>max_depth=3,<br>min_samples_split=2,<br>min_samples_leaf=1,<br>max_features=auto,<br>max_leaf_nodes=None,<br>class_weight=balanced,<br>ccp_alpha=0.02631578947368421 |
| All EMG features | criterion=entropy,<br>splitter=random,<br>max_depth=None,<br>min_samples_split=4,<br>min_samples_leaf=1,<br>max_features=auto,<br>max_leaf_nodes=None,<br>class_weight=balanced,<br>ccp_alpha=0.0 |
| Correlated EMG features | criterion=entropy,<br>splitter=random,<br>max_depth=6,<br>min_samples_split=2,<br>min_samples_leaf=1,<br>max_features=auto,<br>max_leaf_nodes=None,<br>class_weight=balanced,<br>ccp_alpha=0.0 |
| Correlated EMG features and gender | criterion=gini,<br>splitter=random,<br>max_depth=1,<br>min_samples_split=2,<br>min_samples_leaf=1,<br>max_features=auto,<br>max_leaf_nodes=None,<br>class_weight=balanced,<br>ccp_alpha=0.0 |
| Correlated EMG features and age | criterion=gini,<br>splitter=random, |

| Input | Best hyperparameters |
|---|---|
| | max_depth=4,<br>min_samples_split=2,<br>min_samples_leaf=1,<br>max_features=None,<br>max_leaf_nodes=None,<br>class_weight=balanced,<br>ccp_alpha=0.0 |
| Correlated EMG features and body mass | criterion=gini,<br>splitter=random,<br>max_depth=4,<br>min_samples_split=2,<br>min_samples_leaf=1,<br>max_features=None,<br>max_leaf_nodes=None,<br>class_weight=balanced,<br>ccp_alpha=0.0 |
| Correlated EMG features and paretic side | criterion=entropy,<br>splitter=random,<br>max_depth=4,<br>min_samples_split=2,<br>min_samples_leaf=1,<br>max_features=None,<br>max_leaf_nodes=None,<br>class_weight=balanced,<br>ccp_alpha=0.0 |
| Correlated EMG features and age, body mass, and paretic side | criterion=gini,<br>splitter=random,<br>max_depth=4,<br>min_samples_split=2,<br>min_samples_leaf=3,<br>max_features=auto,<br>max_leaf_nodes=None,<br>class_weight=balanced,<br>ccp_alpha=0.0 |
| Correlated EMG features (only gastrocnemius and lateral hamstring muscles) and age, body mass, and paretic side | criterion=entropy,<br>splitter=random,<br>max_depth=3,<br>min_samples_split=2,<br>min_samples_leaf=3,<br>max_features=None,<br>max_leaf_nodes=4,<br>class_weight =balanced,<br>ccp_alpha=0.0 |

| Input | Best hyperparameters |
|---|---|
| Correlated EMG features and all demographic features | criterion=gini, splitter=random, max_depth=6, min_samples_split=2, min_samples_leaf=2, max_features=auto, max_leaf_nodes=8, class_weight=balanced, ccp_alpha=0.0 |
| Correlated EMG features (only gastrocnemius and lateral hamstring muscles) with synthetic white noise samples and age, body mass, and paretic side | criterion=gini, splitter=random, max_depth=4, min_samples_split=2, min_samples_leaf=4, max_features=auto, max_leaf_nodes=8, class_weight=balanced, ccp_alpha=0.0 |
| Correlated EMG features (only gastrocnemius and lateral hamstring muscles) with synthetic pink noise samples and age, body mass, and paretic side | criterion=entropy, splitter=random, max_depth=4, min_samples_split=2, min_samples_leaf=1, max_features=auto, max_leaf_nodes=8, class_weight=balanced, ccp_alpha=0.0 |
| Correlated EMG features (only gastrocnemius and lateral hamstring muscles) with synthetic SMOTE samples and age, body mass, and paretic side | criterion=entropy, splitter=best, max_depth=4, min_samples_split=2, min_samples_leaf=3, max_features=auto, max_leaf_nodes=None, class_weight=balanced, ccp_alpha=0.0 |

Figure S3 shows the training and validation score curves per model complexity (maximum depth of the tree) considering input composed of correlated EMG features (only gastrocnemius and lateral hamstring muscles) with synthetic white noise samples and age, body mass, and paretic side demographic features.

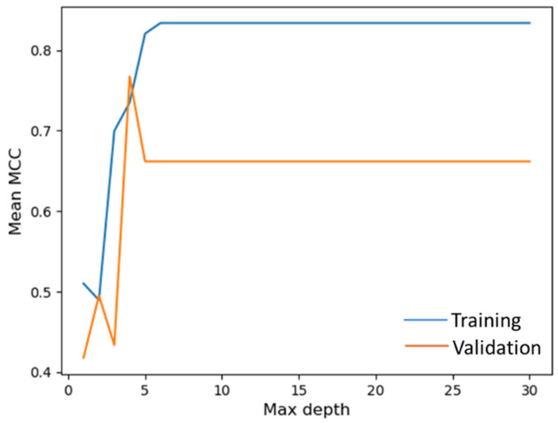


Figure S3. Training and validation MCC score curves per max depth hyperparameter (maximum depth of the tree).

Figure S4 presents the confusion matrix resulting from a) ARRA test set and b) hybrid ARRA + Braga Hospital test sets considering input composed of correlated EMG features (only gastrocnemius and lateral hamstring muscles) with synthetic white noise samples and age, body mass, and paretic side.

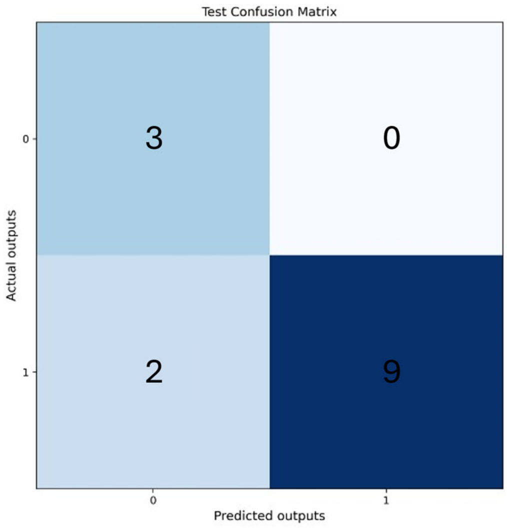


a)

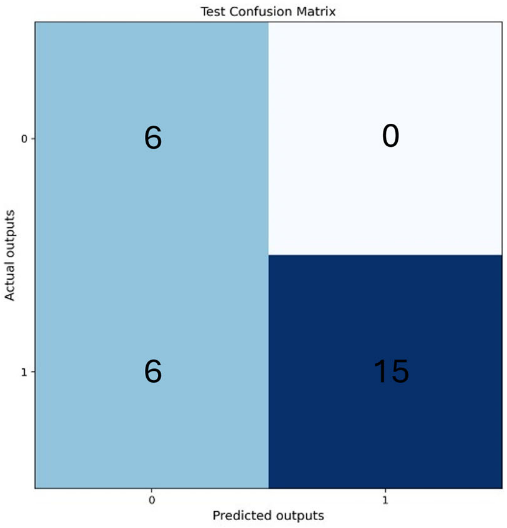


b)

Figure S4. Confusion matrix (low and mid impairment classes labelling 1 and 0, respectively) from a) ARRA test set and b) ARRA + Braga Hospital test sets.

Figure S5 presents the tree diagram resulting from the best hyperparameters found considering input composed of correlated EMG features (only gastrocnemius and lateral hamstring muscles) with synthetic white noise samples and age, body mass, and paretic side.

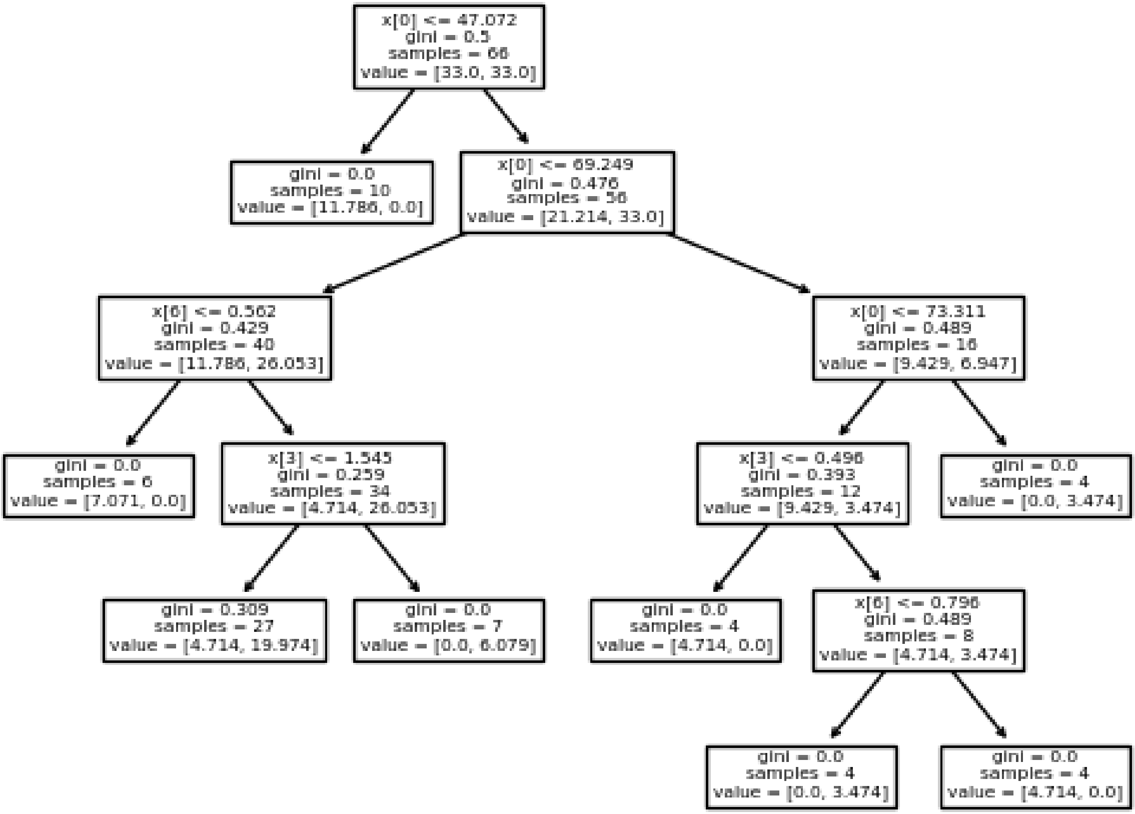


Figure S5. Decision Tree diagram from best hyperparameters using input composed of correlated EMG features (only gastrocnemius and lateral hamstring muscles) with synthetic white noise samples and age, body mass, and paretic side. Order of inputs: age, body mass, paretic side, MDF_G, MDF_LH, PKF_G, PKF_LH.

The age-related decision thresholds identified by the tree should be interpreted cautiously. Although older age is generally associated with poorer post-stroke

outcomes, the present cohort showed age-related patterns that differed from expected epidemiological trends. This may reflect cohort-specific recruitment characteristics, survivor bias, or inclusion criteria requiring independent walking ability.

Table S3. Mean ± standard deviation (*p*-value) values of F1, recall, and MCC scores achieved by decision tree for training and validation datasets using different inputs: all EMG features, moderate to strong correlated (corr.) EMG features, correlated spatiotemporal features, and correlated EMG features combined with demographic features (age, gender, body mass, paretic side). Statistically significant differences between each input and the correlated EMG input (without demographic features) are marked with *. The result with the maximum MCC validation score is highlighted in bold

| Inputs | F1-score Training | F1-score Validation | Recall Training | Recall Validation | MCC Training | MCC Validation |
|---|---|---|---|---|---|---|
| EMG corr. | 0.98 ± 0.01 | 0.88 ± 0.16 | 0.99 ± 0.01 | 0.98 ± 0.04 | 0.92 ± 0.12 | 0.69 ± 0.39 |
| EMG all | 0.99 ± 0.01 (0.71) | 0.83 ± 0.17 (0.44) | 0.99 ± 0.02 (1.00) | 0.88 ± 0.17 (0.28) | 0.98 ± 0.03 (0.71) | 0.64 ± 0.33 (0.56) |
| Spatiotemporal corr. | 0.91 ± 0.03* (0.03) | 0.91 ± 0.13 (0.85) | 0.85 ± 0.07* (0.03) | 0.92 ± 0.19 (0.32) | 0.77 ± 0.06* (0.03) | 0.68 ± 0.36 (1.00) |
| EMG corr. + demographic all | 0.96 ± 0.01* (0.03) | 0.90 ± 0.18 (1.00) | 0.96 ± 0.02 (0.22) | 0.98 ± 0.05 (0.65) | 0.89 ± 0.03* (0.03) | 0.79 ± 0.36 (1.00) |
| EMG corr. + gender | 0.91 ± 0.03* (0.03) | 0.88 ± 0.19 (0.65) | 1.00 ± 0.00 (0.18) | 1.00 ± 0.00 (0.32) | 0.72 ± 0.08* (0.03) | 0.67 ± 0.47 (0.65) |
| EMG corr. + age | 0.97 ± 0.04 (0.22) | 0.87 ± 0.15 (1.00) | 0.94 ± 0.07 (0.14) | 0.92 ± 0.12 (0.18) | 0.92 ± 0.08 (0.22) | 0.75 ± 0.24 (1.00) |
| EMG corr. + body mass | 0.99 ± 0.01 (0.50) | 0.87 ± 0.18 (0.71) | 0.99 ± 0.02 (1.00) | 0.94 ± 0.12 (0.65) | 0.97 ± 0.03 (0.89) | 0.72 ± 0.36 (1.00) |
| EMG corr. + paretic side | 0.98 ± 0.01 (0.44) | 0.86 ± 0.17 (0.46) | 0.97 ± 0.01 (0.22) | 0.93 ± 0.12 (0.18) | 0.95 ± 0.03 (0.31) | 0.70 ± 0.34 (0.71) |
| EMG corr. + age + paretic side + body mass | 0.94 ± 0.03* (0.03) | 0.89 ± 0.17 (0.71) | 0.90 ± 0.06* (0.03) | 0.86 ± 0.18 (0.11) | 0.86 ± 0.07 (0.06) | 0.81 ± 0.27 (0.71) |

| Inputs | F1-score Training | F1-score Validation | Recall Training | Recall Validation | MCC Training | MCC Validation |
|---|---|---|---|---|---|---|
| **EMG corr. (only gastrocnemius, lateral hamstring) + age + paretic side + body mass** | **0.90 ± 0.04 (0.06)** | **0.88 ± 0.19 (1.00)** | **0.84 ± 0.07* (0.04)** | **0.89 ± 0.25 (0.32)** | **0.76 ± 0.09* (0.03)** | **0.84 ± 0.24 (0.59)** |

Table S4. Mean ± standard deviation (*p*-value) values of F1, recall, and MCC scores for training, validation, and test sets (ARRA test set or hybrid test set from ARRA and HB [Hospital of Braga]) using inputs after different data preparation methods: correlated EMG features (only gastrocnemius and lateral hamstring muscles) combined with demographic features (age, body mass, paretic side) and the addition of noisy (white or pink noise) or SMOTE samples. Decision Tree classifier was used. Statistically significant differences between each input and the whitout noise training set are marked with *. The result with the maximum MCC test score is highlighted in bold

| Inputs | F1-score Training | F1-score Validation | Recall Training | Recall Validation | MCC Training | MCC Validation | F1-score Test ARRA | F1-score Test ARRA + HB | Recall Test ARRA | Recall Test ARRA + HB | MCC Test ARRA | MCC Test ARRA + HB |
|---|---|---|---|---|---|---|---|---|---|---|---|---|
| Without noise | 0.90 ± 0.04 | 0.88 ± 0.19 | 0.84 ± 0.07 | 0.89 ± 0.25 | 0.76 ± 0.09 | 0.84 ± 0.24 | 0.62 | 0.67 | 0.45 | 0.57 | 0.40 | 0.06 |
| **White noise** | **0.89 ± 0.03 (0.84)** | **0.85 ± 0.16 (0.46)** | **0.88 ± 0.04 (0.44)** | **0.84 ± 0.23 (0.65)** | **0.73 ± 0.08 (0.84)** | **0.77 ± 0.24 (0.46)** | **0.90** | **0.83** | **0.82** | **0.71** | **0.70** | **0.60** |
| Pink noise | 0.90 ± 0.04 (1.00) | 0.90 ± 0.12 (1.00) | 0.90 ± 0.06 (0.22) | 0.91 ± 0.15 (0.65) | 0.77 ± 0.10 (1.00) | 0.72 ± 0.37 (0.71) | 0.90 | 0.83 | 0.82 | 0.71 | 0.70 | 0.60 |
| SMOTE | 0.98 ± 0.01* (0.03) | 0.93 ± 0.08 (1.00) | 0.98 ± 0.02* (0.04) | 0.98 ± 0.05 (0.65) | 0.94 ± 0.03* (0.03) | 0.77 ± 0.35 (0.46) | 0.88 | 0.87 | 1.00 | 1.00 | 0.00 | 0.00 |
